# Statistical Modeling of HIV, Tuberculosis, and Hepatitis B Transmission in Ghana

**DOI:** 10.1155/2019/2697618

**Published:** 2019-12-23

**Authors:** Clement Twumasi, Louis Asiedu, Ezekiel N. N. Nortey

**Affiliations:** ^1^School of Mathematics, Cardiff University, Cardiff, UK; ^2^Department of Statistics & Actuarial Science, School of Physical and Mathematical Sciences, University of Ghana, Legon, Accra, Ghana

## Abstract

Most mortality studies usually attribute death to single disease, while various other diseases could also act in the same individual or a population at large. Few works have been done by considering HIV, Tuberculosis (TB), and Hepatitis B (HB) as jointly acting in a population in spite of their high rate of infections in Ghana. This study applied competing risk methods on these three diseases by assuming they were the major risks in the study population. Among all opportunistic infections that could also act within HIV-infected individuals, TB has been asserted to be the most predominant. Other studies have also shown cases of HIV and Hepatitis B coinfections. The validity of these comorbidity assertions was statistically determined by exploring the conditional dependencies existing among HIV, TB, and HB through Bayesian networks or directed graphical model. Through Classification tree, sex and age group of individuals were found as significant demographic predictors that influence the prevalence of HIV and TB. Females were more likely to contract HIV, whereas males were prone to contracting TB.

## 1. Introduction

Human immunodeficiency virus (HIV) and acquired immune deficiency syndrome (AIDS) are a variety of disorders caused by infections with the human immunodeficiency virus [1, 2]. The human body might, therefore, experience challenges in fighting off the disease if the immune system is not strong enough. WHO [3] emphasized that as the viral infection advances, it targets the immune system thereby, intensifying the risk of usual infections such as Tuberculosis as well as other predominant infections and tumours that hardly affect people with working immune systems. Tuberculosis generally affects the lungs, but it is also possible to affect other parts of the body. Infection of other organs can result in different forms of symptoms according to Mandell et al. [4]. Hepatitis B (HB) is also an infectious disease caused by HB virus (HBV) that affects the liver of an individual or causes inflammation of the liver. Consequently, coinfections among these underlying diseases on an individual can be very disastrous since the immune system is greatly affected by both viral and bacterial infections.

Deaths from diseases are mostly attributable to a single cause, while more than two of these underlying infections could act on the same individual. In view of that, several researchers have consequently devised or developed methodologies through competing risks to solve this problem by estimating relevant epidemiological quantities including crude, net, and partial probabilities of death. In addition, Tuberculosis has been confirmed by WHO [3] and other health organizations as the leading cause of death among patients infected with HIV as opposed to Hepatitis B. This study seeks to confirm the conditional dependencies that exist among these three underlying diseases statistically through directed graphical models. Furthermore, the Classification tree model was used to assess significant demographic variables (sex, educational level, age, and marital status) on the prevalence of the three diseases.

## 2. Materials and Methods

The data used for the study were collected from a regional hospital in Ghana since it serves as a major referral center. Competing risk methods were applied on a mortality data extracted from a cohort study of the three underlying diseases (HIV, HB, and TB) across age groups. The data used for the directed graphical model and Classification tree models were obtained from a random sample of 201 patients (with demographic information recorded) who were tested of HIV, TB, and HB, respectively.

### 2.1. Competing Risk

From competing risks, there are three probabilities of deaths which would be estimated using the mortality data across age with respect to HIV, TB, and Hepatitis B. These probabilities are the crude, partial crude, and net probability of death. The terms “risk” and “cause” are synonymous but are slightly different depending on the time of occurrence of the condition. Before death, the condition is considered as a risk, but after death, that same condition can be classified as a cause. The definitions of the underlying mortality probabilities as used in this study are as follows:

 
*Crude probability* is the probability of death from a specific cause in the presence of all other risks acting in a population.
 
*Partial crude probability* is the probability of death from specific cause when another risk(s) is eradicated or eliminated from the population.
 
*Net probability* is the probability of death if a specific risk is the only risk in effect in the population.

### 2.2. Application of Competing Risks to Mortality Data

In a human population, estimation of the net and partial crude probabilities is difficult, so they are computed using their relationship with the crude probability of death. The major assumptions were made such that the individuals who died were able to live at least a fraction of 0.5 within the study period and these three underlying diseases were the main risks in the study population. Also, an important information about the proposed Competing risk method by Chiang [5] is that the method is applicable on uncensored mortality data.

Now, let *n*_*i*_=*x*_*i*+1_ − *x*_*i*_ be the length of study period, *P*_*i*_ be the midyear population, *D*_*i*_ be the number of deaths occurring during the study period, *a*_*i*_ denote the average fraction of the interval lived by each of the *D*_*i*_ individuals, and *N*_*i*_ represent the number of individuals alive at *x*_*i*_ among whom *D*_*i*_ deaths occurred.

Then, the age-specific death rate is given by

(1) $$\begin{array}{c} M_{i}=\frac{D_{i}}{P_{i}}. \end{array}$$

The probability of dying in the interval (*x*_*i*_, *x*_*i*+1_) is estimated as follows:

(2) $$\begin{array}{c} {\hat{q}}_{i}=\frac{D_{i}}{N_{i}}, \end{array}$$

(3) $$\begin{array}{c} N_{i}=\frac{\left[D_{i}+\left(1-a_{i}\right)n_{i}D_{i}\right]}{n_{i}}. \end{array}$$

Hence, it can be inferred from equations (1)–(3) that

(4) $$\begin{array}{c} {\hat{q}}_{i}=\frac{n_{i}M_{i}}{1+\left(1-a_{i}\right)n_{i}M_{i}}, \end{array}$$

(5) $$\begin{array}{c} {\hat{p}}_{i}=\frac{1-a_{i}n_{i}M_{i}}{1+\left(1-a_{i}\right)n_{i}M_{i}}. \end{array}$$

Since ${\hat{q}}_{i}+{\hat{p}}_{i}=1$, the number of deaths occurring during the study period (*D*_*i*_) can be categorized according to the cause (HIV, TB, and HB) with *D*_*iσ*_ dying from cause *R*_*σ*_, *σ*=1,2,3. This implies

(6) $$\begin{array}{c} D_{i}=D_{i1}+D_{i2}+D_{i3}, \end{array}$$

so

(7) $$\begin{array}{c} M_{i\delta }=\frac{D_{i\delta }}{P_{i}}, \end{array}$$

is the cause-specific death rate such that

(8) $$\begin{array}{c} D_{i\delta }=M_{i\delta }\times P_{i}. \end{array}$$

Hence, the crude probability of death is estimated as

(9) $$\begin{array}{c} {\hat{Q}}_{i\sigma }=\frac{n_{i}M_{i\sigma }}{1+\left(1-a_{i}\right)n_{i}M_{i}}, \end{array}$$

and the net probability of dying is given as

(10) $$\begin{array}{c} {\hat{q}}_{i\sigma }=1-{\hat{p_{i}}}^{\left(D_{i\sigma }/D_{i}\right)}. \end{array}$$

The partial crude probability is given by

(11) $$\begin{array}{c} {\hat{Q}}_{i\sigma \cdot 1}=\frac{D_{i\sigma }}{D_{i}-D_{i1}}\left[1-{\hat{p_{i}}}^{\left(D_{i}-D_{i1}/D_{i}\right)}\right]. \end{array}$$

### 2.3. Bayesian Networks

Bayesian network learned via score-based methods (Hill climbing algorithm) was used to determine the joint distribution, marginals, as well as the conditional dependencies that exist among these three underlying diseases. Bayesian network is a probabilistic graphical model that represents a set of random variables and their conditional dependencies via directed acyclic graphs.

**Theorem 1 .**

Let *G*=(*V*, *E*) be directed acyclic graph (DAG) and let *X* = (*X*_*v*_)_*vεV*_ be a set of random variables indexed by *V*. *X* is said to be a Bayesian network with respect to *G* if it satisfies the local Markov property such that each variable is independent of its nondescendants given its parent.

Given any set of random variables (*X*_1_, *X*_2_,…, *X*_*n*_) that satisfies the local Markov property, then its Bayesian network can be represented generally in probability settings as

(12) $$\begin{array}{c} P\left(X_{1},X_{2},\ldots ,X_{n}\right)=\overset{n}{\underset{i=1}{\prod }}P\left(X_{i}\left|X_{pa\left(i\right)}\right.\right), \end{array}$$

where *pa*(*i*) are the parents of node *i*. From the joint distribution, the marginal distribution or any conditional probability can be estimated using either the Naive method or Belief Propagation method.

### 2.4. Classification Tree

The Classification (decision) tree is a type of controlled learning algorithm (having a predefined target variable) that is frequently implemented in problems of classification. It operates on either categorical or continuous variables (both outcome and predictor variables). With this technique, splittings are done by dividing underlying variable into two or more homogeneous sets (or subpopulations) based on most significant variable or splitter. In summary, the decision tree is fitted through supervised learning algorithms by first overfitting the tree, after which it is been pruned using the required complexity parameter associated with the number of nodes with the least relative error if necessary. The relevant terminologies for the Classification Tree method are as follows.

#### 2.4.1. Terminologies of the Decision or Classification Tree

**Figure 1:**
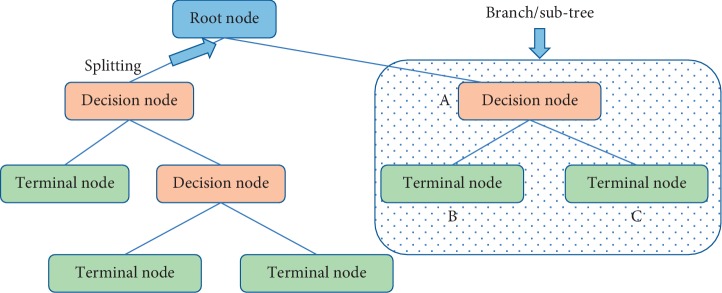
General representation of decision tree.

 
*Node*. It denotes a variable in the tree model.
 
*Root Node*. It represents the most significant variable among all predictor variables, splitting into two homogeneous sets starting at this node.
 
*Splitting*. It is a process of dividing a node into two or more subnodes.
 
*Decision Node*. It is a node that can further be split into subnodes.
 
*Leaf or terminal Node*. A node that cannot split or divide further into subnodes.
 
*Pruning*. It is the removal of subnodes of decision nodes after overfitting into relatively smaller size so as to achieve a better predictive power.
 
*Branch or SubTree*. A subsection of full tree upon splitting of the root node is called branch or subtree.
 
*Parent and Child Node*. A node which is divided into subnodes is called parent node, whereas the subnodes are called the children of parent node.  Figure 1 is a general representation of a decision tree model.

## 3. Result and Discussion

### 3.1. Crude, Net, and Partial Probability Estimation across Age

Table 1 presents estimation of the crude, net, and partial probabilities of death generated from the uncensored mortality data.

**Figure 2:**
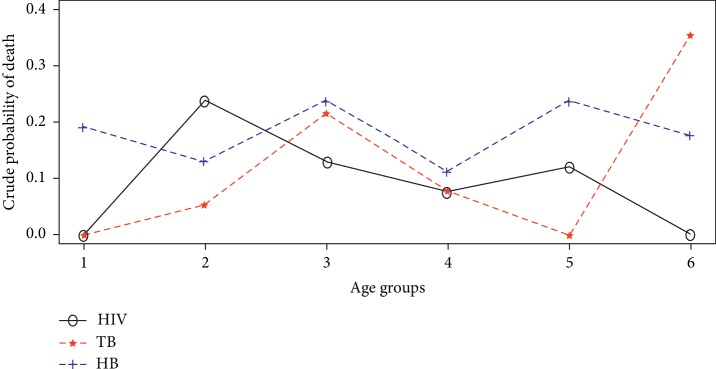
Crude probability of death from the underlying diseases.

The crude probability of death was estimated from equation (9). For instance, in estimating the crude probability of dying from HIV 20–34 age group (as presented in Table 1), *n*_2_=15, *a*=0.5, *D*_2*σ*_=9, *D*_2_=14, *P*_2_=464, and *M*_2_=14/464 such that the crude probability is estimated from (9) as 0.2373.  Figure 2 is a diagrammatic representation of the estimates of the crude probability of deaths for each disease.

It can be observed from Figure 2 that the probability of an individual dying from Hepatitis B in the presence of all other diseases was relatively higher than that of Tuberculosis within the study population across age groups except at the age interval of 70 to 79 years (represented as 6). However, the crude probability of death from HIV was only higher than that of TB and HB at age group 20–34 years (represented as 2) and generally lower than the crude probability estimates for HB at higher age intervals. Also, the probability of dying from HIV was found to be higher than that of TB at age interval 60 to 69 years, but averagely lower at all other age intervals. It can be inferred that the probability of an individual dying from Hepatitis B across age was comparatively higher on the average than dying from either HIV or TB when all other diseases acting in the study population or cohort of individuals considered.

**Figure 3:**
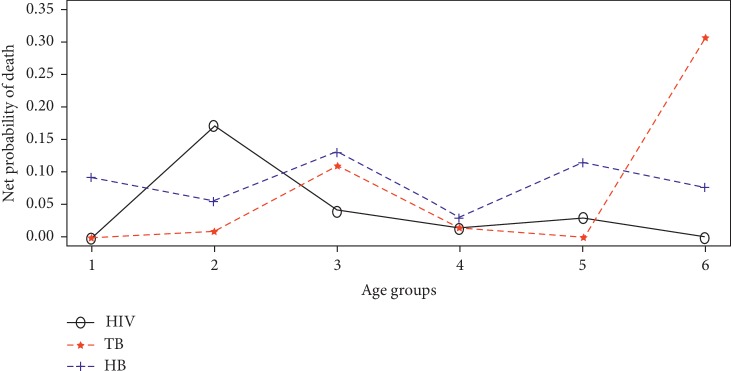
Net probability of death from the underlying diseases.

Also, the net probability of death was considered as the probability death if a specific risk (HIV, TB, or HB) was the only risk acting in the study population. Equation (10) was used to compute the estimates of the net probabilities of death. For instance, to estimate the net probability of dying from HIV for age group 2 (20–34 years), *n*_2_=15, *a*=0.5, *D*_2*σ*_=9, *D*_2_=14, *P*_2_=464, *M*_2_=14/464, and from equation (5), ${\hat{p}}_{2}=359/569$. Hence, from equation (10), the net probability is estimated as 0.1717.  Figure 3 shows a combined graph of the net probabilities for each disease.

From Figure 3, the net probability of deaths from HIV and TB were higher than that of Hepatitis B at age intervals 20–34 years (denoted as 2) and 70–79 years (denoted as 6), respectively. However, the probability of an individual dying from Hepatitis B if it was the only risk in effect was higher than that of HIV and TB at all other age intervals. Additionally, the net probability of deaths from TB was also higher than that of HIV at all age intervals except at 20–34 years and 60–69 years. These findings suggest that Hepatitis B was on the average more infectious followed by Tuberculosis. It can also be inferred that the probability of dying from a specific risk or disease (HIV, TB, or HB) is contingent on the age of the infected individual.

**Figure 4:**
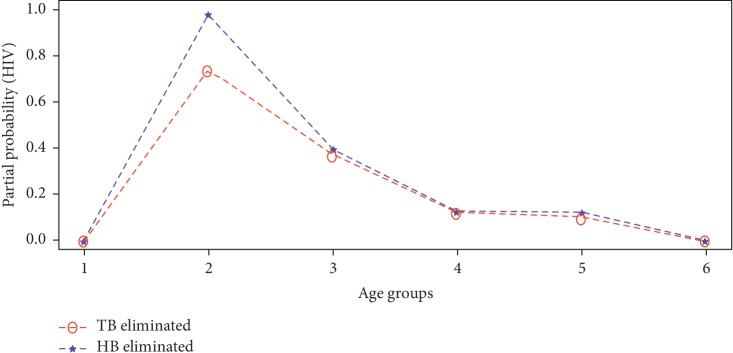
Partial probability of death from HIV.

**Figure 5:**
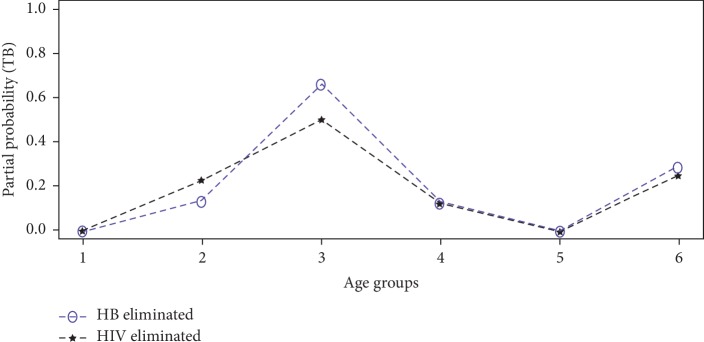
Partial probability of death from Tuberculosis.

**Figure 6:**
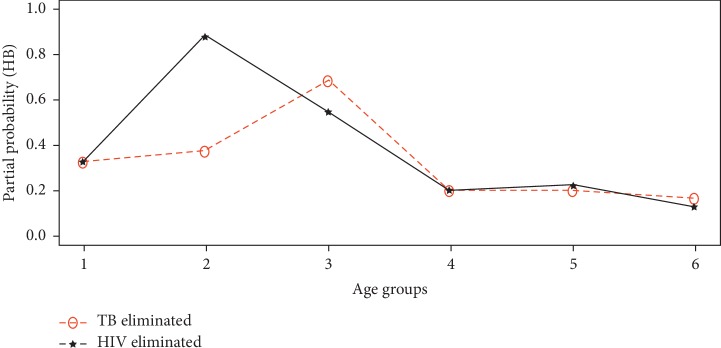
Partial probability of death from Hepatitis B.

The partial probability of death was computed for each disease from equation (11) upon eliminating HIV, TB, or HB from the study population. The estimates of the partial probability of death from HIV, TB, and HB are presented by Figures 4–6, respectively.

It can be deduced from Figure 4 that the probability of an individual dying from HIV if Hepatitis B is eliminated from acting in the study population is relatively higher than when Tuberculosis is eliminated at all age intervals. Also, it can be seen from Figure 5 that the probability of an individual dying from Tuberculosis if Hepatitis B is eliminated as a risk is higher for age group 35 to 49 years. On the contrary, Figure 6 revealed that the partial probability of dying from Hepatitis B if HIV or Tuberculosis was removed from acting in the study population was higher at age interval 20–34 years and 35–49 years, respectively. Generally the partial probabilities of deaths were relatively higher at age intervals 20–34 years and 35–49 years. This means, on the average, individuals between the ages 20 and 50 years were at a greater risk of dying from any of these three underlying diseases within the study population as opposed to other age intervals.

### 3.2. Bayesian Network Application

Bayesian network learned via score-based methods (Hill climbing algorithm) was used to determine the joint distribution, marginals, as well as the conditional dependencies that exists among these three underlying diseases. Positive and negative test results were quantified as {1} and {0}, respectively.

### 3.3. Structure Learning

**Figure 7:**
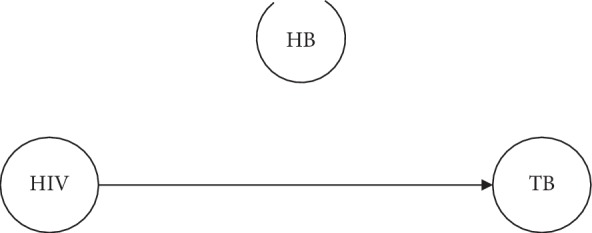
Directed acyclic graph of the three diseases.

The directed paths (arcs or edges) between any pair of nodes (diseases) were found by determining any node that d-separates one node from the other. However, the node that d-separate the two other nodes simply blocks every undirected path between the two nodes, thereby making the underlying two nodes conditionally independent of each other. It was realized that HIV and Tuberculosis are conditionally dependent or related given Hepatitis B and can be inferred that individuals with HIV mostly contract Tuberculosis as opposed to Hepatitis B. This statistically confirmed why TB is predominant among HIV patients as opposed to Hepatitis B.  Figure 7 shows the Bayesian network of HIV, TB, and HB.

Table 2 presents the estimates of the marginal distribution for HIV and Hepatitis B.

It can be seen from Table 2 that the probability of an individual getting infected with HIV is approximately 0.61, whereas the probability of that same individual experiencing Hepatitis B infection is approximately 0.13.

**Figure 8:**
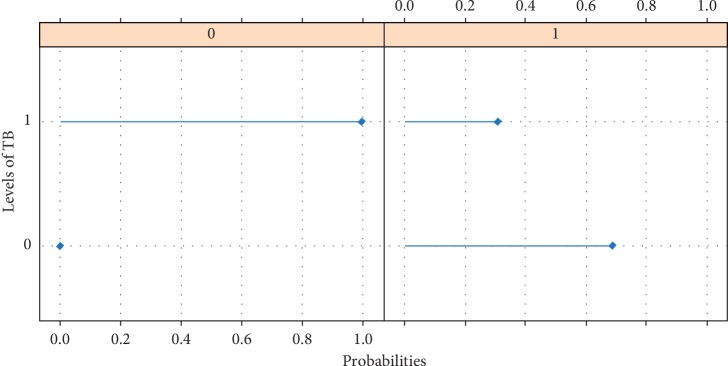
Graph of conditional distribution of TB infection given HIV.

Figure 8 presents the conditional distribution of TB given HIV. It was discovered that an individual with HIV has a probability of 0.69 of not contracting TB and a probability of 0.31 of contracting TB. It is also certain that an individual without HIV can easily contract TB.

### 3.4. Classification Tree Model

The effects of sociodemographic characteristics on the prevalence of these diseases within the study population were, respectively, assessed. In determining the significant demographic factors, the Classification tree model was employed. Sex (*p* < 0.001) and age (*p* < 0.001) of patients were found to be the most significant demographic factors that influenced the prevalence of diseases (HIV and TB) from both models within the cohort.

#### 3.4.1. Fitting the Classification Tree Model

**Figure 9:**
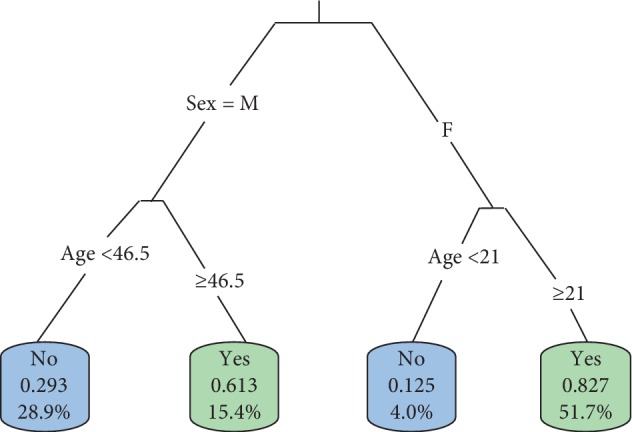
Fitted tree model for the prevalence of HIV.

**Figure 10:**
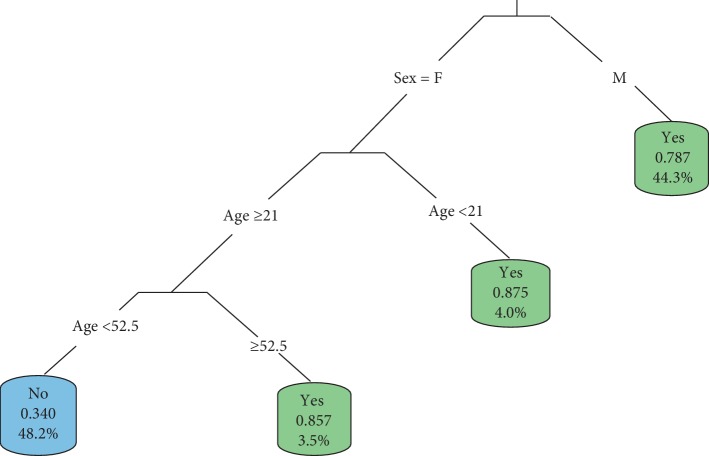
Fitted tree model for the prevalence of Tuberculosis.

In identifying the best size of the tree, the model was overfitted first using the most significant variables at each stage of splitting, after which it was pruned. The tree model using Hepatitis B as a dependent variable was not presented since none of the independent variables had significant effect on the prevalence of HB. Figures 9 and 10 show the fitted tree model for the prevalence of HIV and TB, respectively.

It can be inferred from the fitted tree model that male patients whose age was 46.5 years and above were likely to be infected with HIV, while female patients who were 21 years and above were more susceptible to HIV infection. In addition, the tree model classified approximately 51.7% of females of at least 21 years within the study population to be HIV positive, whereas 15.4% of males at least 46.5 years were classified HIV positive. Hence, it can be inferred that females are likely to contract HIV than males at early age. The high incidence of HIV among women is mostly due to intimate partner violence or sexual abuse [6]. Also, females above 20 years also contract HIV as opposed to those less than 20 years. According to UNAIDS [7], this could be due to financial disparities and intimate partner violence in relationships which often prevent women (especially in Africa) from negotiating on condom use.

Also, it can be deduced from Figure 10 that males developed TB at any time irrespective of their age. The tree model classified about 44.3% of males to be TB positive. Females were more likely to contract TB at ages either less than 21 years or at least 52.5 years. Among females less than 21 years, approximately 4.0% were classified by the tree model as TB positive, whereas 3.5% of females at least 52.5 years were found to be TB positive. However, females between 21 and 52.5 years were likely to be free from Tuberculosis within the study population. Males found to be more likely to develop TB relative to females and are in consonance with findings from other studies [8]. In their study, they found out that nearly twice as many men as women have been diagnosed with TB globally and the imbalance in incidence is usually attributed to social, cultural, and economic factors. Responsibility of men in the community may also require them to have more social contact, thereby increasing the risk of TB exposure as asserted by Liefooghe et al. [9] and Vlassoff and Moreno [10].

## 4. Conclusion and Recommendation

The competing risk methods revealed that individuals of the study population between the ages of 20 and 50 years had greater chance of dying from these three HIV, HB, and TB. In addition, the Classification tree discovered that females were likely to contract HIV relative to males, whiles males were rather prone to contracting Tuberculosis comparatively. Also, TB was found to be very prevalent among HIV-infected individuals compared with Hepatitis B from the fitted directed graphical model.

Sex and age of patients were found to be the significant demographic variables that contributed to the prevalence of HIV and TB in the study population as opposed to marital status and educational level. But, none of the demographic characteristics influenced Hepatitis B prevalence unlike HIV and TB. It was also found that 51.7% of females at least 21 years within the study population were HIV positive, whereas 15.4% of males at least 46.5 years were classified HIV positive. Also, it categorized about 44.3% of males to be TB positive. Among females less than 21 years, approximately 4.0% were classified by the tree model as TB positive, whereas 3.5% of females at least 52.5 years were found to be TB positive.

From these findings, it was recommended that public health education and other symposiums should be organized for both males and females on the prevalence of these underlying diseases so as to create high level of awareness about them. Frequent immunization against Tuberculosis among HIV patients is also recommended.

## Figures and Tables

**Table 1:** Current mortality data of the three diseases across age.

Age	Cases diagnosed	Total deaths	HIV deaths	TB deaths	HB deaths
15–19	58	8	0	0	3
20–34	464	14	9	2	5
35–49	499	26	6	10	11
50–59	184	16	2	2	3
60–69	34	10	1	0	2
70–79	16	8	0	2	1

**Table 2:** Marginal probability distributions.

Variable	No	Yes
HIV	0.3880597	0.6119403
Hepatitis B	0.8656716	0.1343284

## Data Availability

The data used to support the findings of this study are available from the corresponding author upon request via the email address lasiedu@ug.edu.gh.
